# Occurrence and pathogenicity of *Corinectria* spp. – an emerging canker disease of *Abies sibirica* in Central Siberia

**DOI:** 10.1038/s41598-020-62566-y

**Published:** 2020-03-27

**Authors:** Igor N. Pavlov, Rimvydas Vasaitis, Yulia A. Litovka, Jan Stenlid, Libor Jankovsky, Anton A. Timofeev, Audrius Menkis

**Affiliations:** 1 V.N. Sukachev Institute of Forest SB RAS, Laboratory of Reforestation, Mycology and Plant Pathology, Krasnoyarsk, 660036 Russia; 2 Reshetnev Siberian State University of Science and Technology, Department of Chemical Technology of Wood and Biotechnology, Krasnoyarsk, 660037 Russia; 3 Swedish University of Agricultural Sciences (SLU), Department of Forest Mycology and Plant Pathology, P.O. Box 7026, SE-75007 Uppsala, Sweden; 4 Mendel University in Brno, Department of Forest Protection and Wildlife Management Zemedelska 3, 61300 Brno, Czech Republic

**Keywords:** Environmental microbiology, Fungi, Forestry, Invasive species

## Abstract

During recent years, a new disease of Siberian fir (*A. sibirica*) emerged in Central Siberia, exhibiting symptoms of stem/branch deformation, cambium necrosis, and dieback of branches and twigs, the causal agent remaining unknown. The aim was to identify agent of the disease and to investigate its pathogenicity to *A. sibirica* and Norway spruce (*Picea abies*). Symptomatic tissues of fir were subjected to pure culture isolation of anticipated pathogen(s). Obtained isolates were subjected to molecular identification, phylogenetic analyses, and pathogenicity tests with *A. sibirica* saplings, and seeds and seedlings of *A. sibirica* and *P. abies*. The study demonstrated that, (i) most commonly isolated fungus from canker wounds of *A. sibirica* exhibited *Acremonium-*like anamorphs; (ii) phylogeny demonstrated that investigated fungi belong to genus *Corinectria*, but are genetically well separated from other worldwide known *Corinectria* spp.; (iii) one species of isolated fungi has the capacity to cause the disease and kill *A. sibirica* saplings and seedlings, but also seedlings of *P. abies*. Guidelines for future research were defined in order to generate needed information on species description, its origin and ecology, and estimation of potential risks upon the eventual invasion of the pathogen to new geographic areas, in particular of Europe.

## Introduction

**Figure 1:**
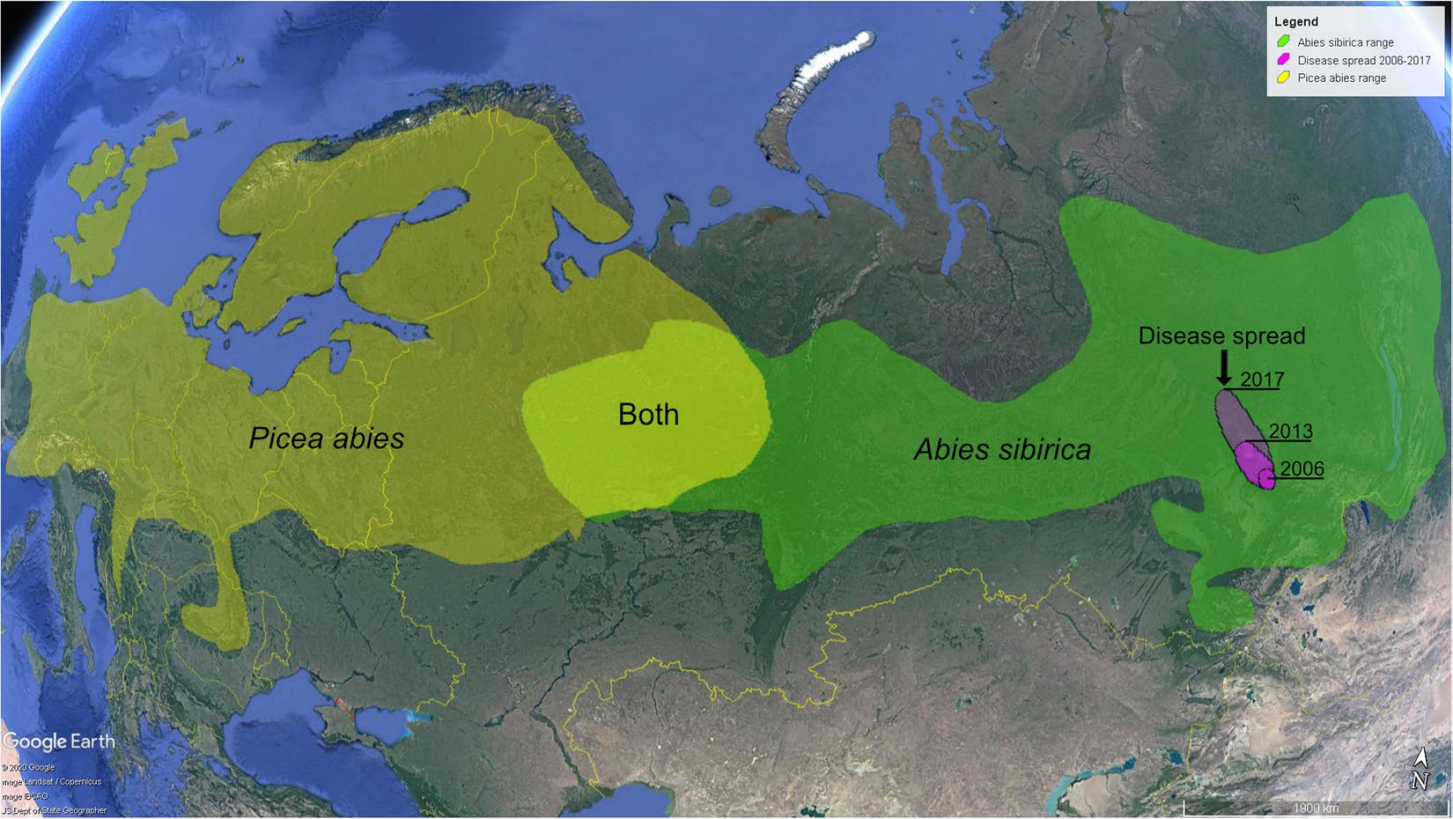
Map of Eurasia showing distribution range of *Picea abies* (dark yellow), *Abies sibirica* (green), and in common for both tree species (light yellow). Spread of the disease during 2006–2017 is shown in purple. The map was produced using Google Earth Pro v. 7.3.2.5776 available at www.google.com/earth/.

*Abies sibirica* Ledeb., the Siberian fir, is a very widespread tree species: the range of its native distribution covers a huge geographic area, stretching from Northern Europe – Russia (starting east of the Volga River), through Siberia to northern China, Mongolia, and the Russian Far East, reaching north up to 67°40′ Northern latitude (http://www.agroatlas.ru/en/content/related/Abies_sibirica/map/index.html) (Fig. 1). There, *A. sibirica* forms extensive forests on the northern plains and on cool wet mountainsides, growing on soils that are usually of alluvial origin, podzolic, calcareous, well drained and free of permafrost; in the mountains, reaching elevations up to 2000 metres. It is an economically important timber species, extensively harvested from vast natural stands for use in construction, pulp etc.; the tree also has local medicinal uses^1^. Notably, native range of *A. sibirica* in the European part of Russia (west of Ural Mountains reaching the Volga River) overlaps with that of Norway spruce (*Picea abies* (L.) Karst.) (Fig. 1), one of the most economically important timber producing tree species of Europe.

**Figure 2:**
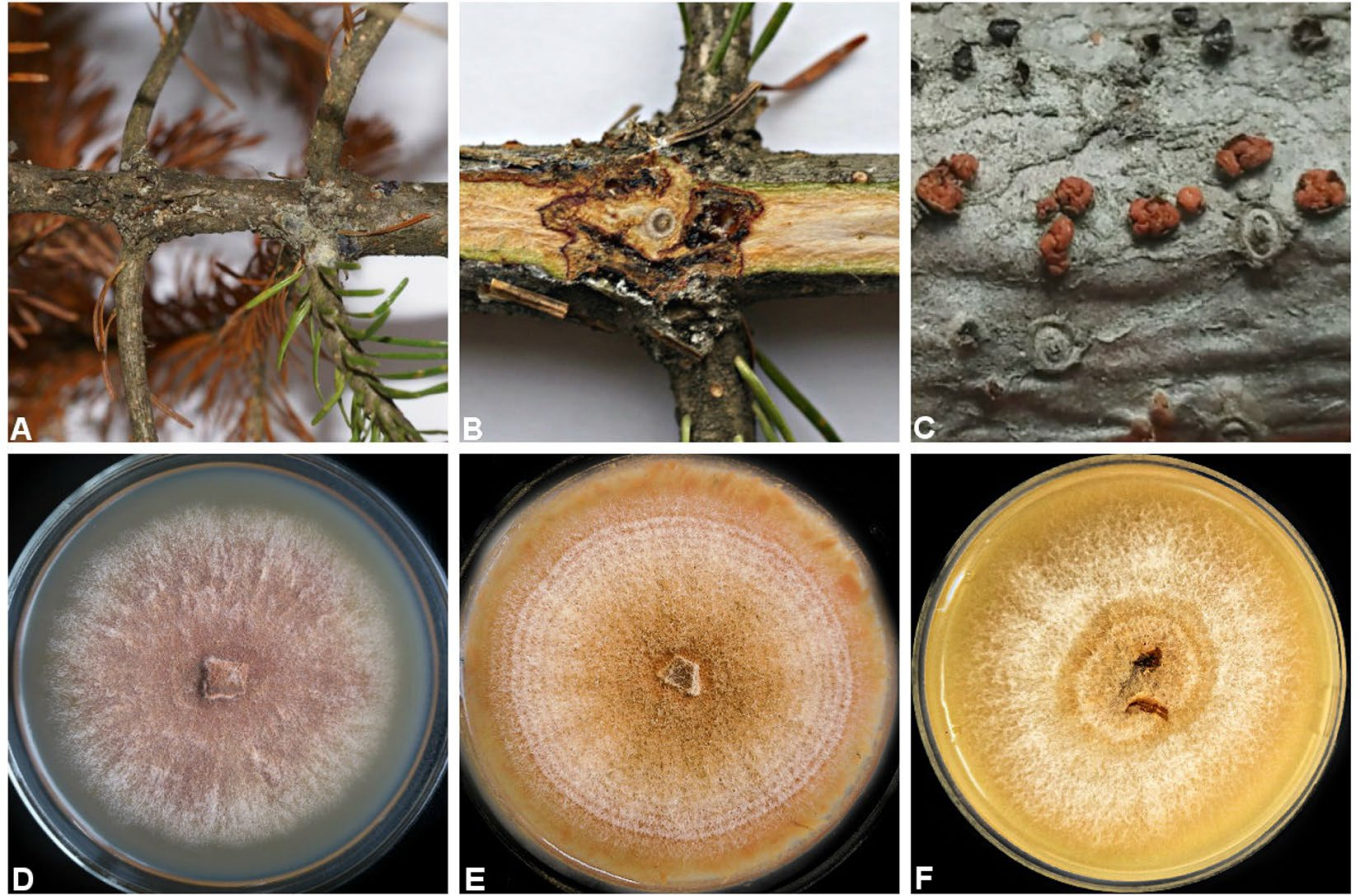
Typical symptoms and signs of investigated fungus (presumably *Corinectria* spp.) on approx. 5-10 mm diameter branch of *Abies sibirica* (**A,B**); red fungal fruitbodies (**C**) (approx. 1 mm in diameter); colony of the fungal strain N1 (NfP5.7) on PDA (**D**), carrot-agar (**E**) and MEA (**F**).

During recent years, a new disease of *A. sibirica* emerged in Central Siberia (Fig. 1), exhibiting symptoms of stem/branch deformation, cambium necrosis, and dieback of branches and twigs, with the subsequent development on those of round red fungal fruitbodies (Fig. 2A–C). The disease is typically observed on relatively young re-growth trees, and often results in their death.

The first symptoms of the disease have been observed in the year 2006 in the eastern Sayan Mountains (N54°29′13″; E93°27′11″; elevation 700 m), and after following two years it was repeatedly observed within the approx. the same geographic range (N54°43′05″; E93°26′40″), but then on higher elevations with a colder climate. In year 2010, the disease has expanded approx. 160 km northwards, emerging in the vicinity of Krasnoyarsk City (N56°01′17″; E92°34′05″; elevation 270 m). In later years, starting with 2013, characteristic symptoms started to appear further north, on higher elevations in South Siberia (N56°08′46″; E92°11′58.77″; elevation 500 m). In year 2017, the disease spread 300 km northwards, and has reached the geographic range of N58°49′52″; E 93°10′19″ (elevation 400 m). Consequently, during eleven years starting from its initial observation (2006–2017), the disease spread northwards over geographic distance exceeding 450 km (Fig. 1 and internal reports of V.N. Sukachev Institute of Forest Siberian Branch, Russian Academy of Science).

Despite the similarity of symptoms caused, the resemblance of the sporocarps, and pure culture morphology (Fig. 2) to those of conifer canker fungus *Corinectria* (prev. *Neonectria*) *fuckeliana* (C. Booth) C. Gonzalez & P. Chaverri^2,3^, the identity and origin of this (possibly related) pathogen observed on diseased *A. sibirica* in Central Siberia was remaining unknown. A key to *Corinectria* species is provided by Gonzalez and Chaverri^2^. The aim of the present study was to identify the causal agent of the disease and to investigate its pathogenicity to *A. sibirica* and, preliminary, to *P. abies*.

## Results

### Isolation of fungi

**Figure 3:**
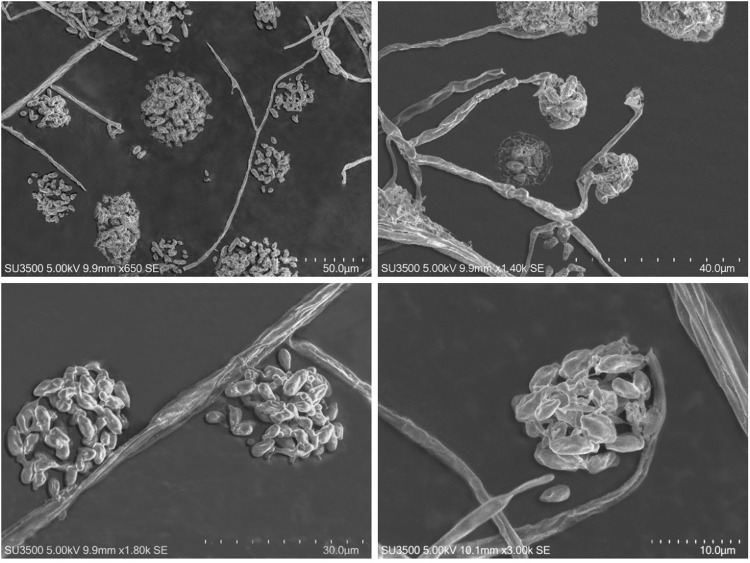
Scanning electron microscopy of conidiophores and microconidia (magnification x 650–3000 times) of the *Acremonium* – like anamorph fungal strain N1 (NfP5.7).

In total, 47 distinct/different morphotypes of pure cultures of fungi were isolated (data not shown). Pure culture morphotype representing *Acremonium-*like anamorph (Fig. 2D–F; Fig. 3) was isolated from 14 out of 64 cankers (phloem/xylem/periderm) that were subjected to isolation (22%), and was the most frequently isolated fungus. In more detail, isolation frequencies and respective numbers of obtained *Acremonium*-like isolates from different canker types are presented in the Table 1, which demonstrates that *Acremonium*-like isolates has been consistently isolated (although with varying frequency of 17–30%) from each canker type. On different agar media, isolated *Acremonium*-like isolates varied in colour that was light purple (Fig. 2D), light orange (Fig. 2E) or light yellow (Fig. 2F). The rest of the isolates were not identified.

**Table 1:** Sampled *Abies sibirica* cankers and frequency of *Acremonium-*like anamorph (*Corinectria*) isolations.

Isolations	No./size^a,b,c^ of sampled cankers and no. (%) of isolates obtained			
	branch cankers^a^ (n = 31)	small stem cankers^b^ (n = 27)	large stem cankers^c^ (n = 6)	all (n = 64)
*Acremonium-*like anamorph	5 (16%)	8 (30%)	1 (17%)	14 (22%)
Other fungi	17 (55%)	14 (52%)	2 (33%)	33 (52%)

^a^approx. size: length × width, 10 × 6 mm;

^b^approx. size: length × width, 35 × 18 mm;

^c^approx. size: length × width, 120 × 50 mm.

### Molecular identification and phylogenetic analyses

**Figure 4:**
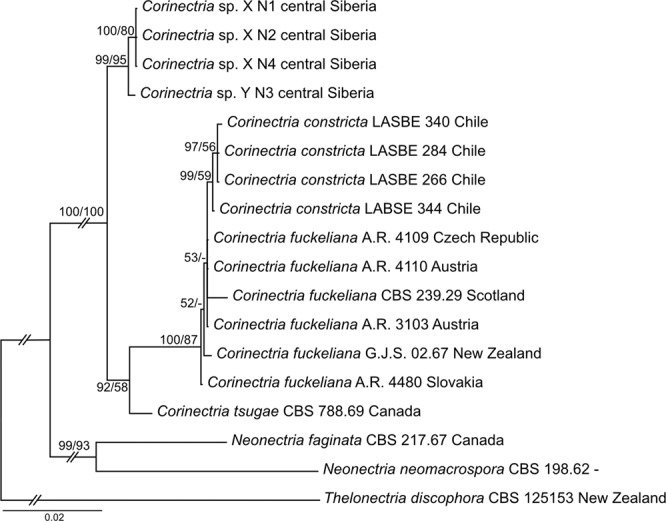
Phylogeny of several Nectriaceae taxa showing the Bayesian Inference best tree inferred from four genes (ACT, BTUB, ITS & TEF1, but not all sequences were available for all taxa). Support values are indicated at the nodes (BI posterior probabilities/ML bootstrap). Outgroup taxa is *Thelonectria discophora*. The results of the BI and ML analyses showed a strong support for the clade that includes the Siberian isolates, which are clearly separated from the other *Corinectria* spp. Siberian isolates were subdivided into two clades; three isolates that clustered together were denoted by X and a single isolate – by Y. The strain of *C. tsugae* is a type strain.

For the phylogenetic analyses, four *Acremonium*-like Siberian isolates, four isolates of *Corinectria constricta*, six isolates of *C. fuckeliana*, and an isolate of each C*. tsugae*, *Neonectria faginata*, *N. neomacrospora* and *Thelonectria discophora* (Table 2) were included into the multi-gene (ACT, BTUB, ITS and TEF1) alignment that was 2555 bp in length with 173 parsimony-informative sites and 285 variable parsimony-uninformative sites. Analyses showed that four Siberian isolates formed a sepatare clade within *Corinectria* that was well supported by both BS and posterior probability (Fig. 4). In addition, the Siberian isolates were subdivided into two well-supported clades. One clade included three isolates denoted by *Corinectria* sp. X and the other clade included a single isolate denoted by *Corinectria* sp. Y. The phylogeny results demonstrate that investigated Siberian *Corinectria* spp., despite being most closely related to this genus, are genetically well separated from other known *Corinectria* spp. from around the globe, and that Siberian *Corinectria* sp. X and sp. Y likely represent two different taxa on their own. Among the three isolates of *Corinectria* sp. X there was no sequence difference in all loci used. Comparison between *Corinectria* sp. X and sp. Y showed that there was 2 bp difference in ACT, 2 bp difference and 3 bp insertion in BTUB, 1 bp difference and 1 bp deletion in ITS, and 1 bp difference in TEF1.

**Table 2:** Isolates used in the present study and their GenBank accession numbers.

Species	Isolate/voucher^a^	Geographic origin	Host	GenBank accession number			
				ACT	BTUB	ITS	TEF1
*Corinectria* sp. X	N1 (NfP5.7)	Central Siberia	*Abies sibirica*	MN684339	MN684343	MN655987	MN684347
*Corinectria* sp. X	N2 (NfSp3.4)	Central Siberia	*Abies sibirica*	MN684340	MN684344	MN655988	MN684348
*Corinectria* sp. X	N4 (NfP5.5)	Central Siberia	*Abies sibirica*	MN684342	MN684346	MN655990	MN684350
*Corinectria* sp. Y	N3 (N2.1-18)	Central Siberia	*Abies sibirica*	MN684341	MN684345	MN655989	MN684349
*Corinectria constricta*	LASBE 266; RGM 2382; SGO 167410	Chile	*Pinus radiata*	KY636427	KY636417	—	KY636410
*C. constricta*	LASBE 284; SGO 167411	Chile	*Pinus radiata*	KY636428	KY636418	—	KY636411
*C. constricta*	LASBE 340; SGO 167416	Chile	*Pinus radiata*	KY636433	KY636423	—	KY636414
*C. constricta*	LASBE 344; SGO 167417	Chile	*Pinus radiata*	KY636434	KY636424	—	KY636415
*Corinectria fuckeliana*	A.R. 3103; BPI 842140	Austria	*Picea abies*	HM352872	HM352857	HM364291	HM364342
*C. fuckeliana*	A.R. 4109; CBS 119723; IMI 871121; BPI 871121	Czech Republic	*Pinus sylvestris*	HM352873	HM352858	HM364292	HM364343
*C. fuckeliana*	A.R. 4110; CBS 119200; IMI 871034	Austria	*Picea abies*	HM352874	HM352859	HM364293	HM364344
*C. fuckeliana*	A.R. 4480; IMI 879930	Slovakia	Dead bark	HM352876	HM352861	HM364295	HM364346
*C. fuckeliana*	CBS 239.29	Scotland	*Picea sitchensis*	—	DQ789871	HQ840386	JF268748
*C*. cf. *fuckeliana*	G.J.S. 02.67	New Zealand	*Pinus radiata*	HM352886	HM352867	HM364300	HM364354
*Corinectria tsugae*	CBS 788.69	Canada	*Tsuga heterophylla*	—	KM232020	KM231763	—
*Neonectria faginata*	CBS 217.67	Canada	*Fagus grandifolia*	KC660412	—	HQ840385	JF268746
*N. neomacrospora*	CBS198.62	—	*Abies concolor*	—	HM352865	AJ009255	HM364351
*Thelonectria discophora*	CBS 125153; A.R. 4324	New Zealand	*Pinus radiata*	HM352875	—	HM364294	KM231897

^a^*A.R*. Collection of A.Y. Rossman; *BPI* The U.S. National Fungus Collections; *CBS* CBS-KNAW culture collection, Westerdijk Fungal Biodiversity Institute, Utrecht, The Netherlands; *G.J.S*. Collection of Gary J. Samuels; *IMI* International Mycological Institute, CABI-Bioscience, Egham, Bakeham Lane, UK; *LASBE* Fungus Collection, Laboratorio de Salud de Bosques y Ecosistemas, Universidad Austral de Chile, Valdivia, Chile; *RGM* Chilean Collection of Microbial Genetic Resources; *SGO* Herbarium SGO, Chilean National Museum of Natural History, Santiago, Chile.

### Pathogenicity tests

Results of pathogenicity tests are presented in the Table 3.

**Table 3:** Results of pathogenicity tests. Chi-squared tests were used to estimate the differences in proportions, and *t*-tests were used to estimate the differences between means.

Parameter	Isolate N1 (NfP5.7)	Isolate N2 (NfSp3.4)	Pooled N1 + N2	Control
**Saplings of** ***Abies sibirica***				
Tested (no.)	30	30	60	30
Dead & diseased (no./%)				
– dead	7/23.3	5/16.7^a^	12/20.0	2/6.7^c^
– diseased, phloem necrotic	20/66.7	21/70.0^a^	41/68.3	0/0^d^
Visually healthy (no./%)	3/10.0	4/13.3^a^	7/11.7	28/93.3^d^
Phloem necrosis (mm, mean ± SD)				
– longitudinal	46.9 ± 3.0	41.9 ± 2.5^b^	44.4 ± 2.8	0 ± 0^d^
– lateral at cross-section	18.2 ± 0.8	15.9 ± 0.6^b^	17.1 ± 0.7	0 ± 0^d^
Re-isolation of the fungus (no./%)	24/80.0	22/73.3	46/76.7	—
**Seeds and seedlings of** ***Abies sibirica***				
Tested (no.)	300	300	600	300
Germinated seeds (no./%)	199/66.3	203/67.7^a^	402/67.0	210/70.0^c^
Dead & diseased (no./% of germinated)	169/84.9	165/81.3^a^	334/83.1	18/8.6^d^
Chip coverage by mycelium (no. chips checked/mean score ^e^)	45/2.2	45/1.9	90/2.1	—
Re-isolation of the fungus from				
– chips (no. checked/no. isolated/%)	24/22/91.7	24/20/83.3	48/42/87.5	—
– inoculated seedlings (no./%)	156/92.3	147/89.1	303/90.7	—
**Seeds and seedlings of** ***Picea abies***				
Tested (no.)	300	300	600	300
Germinated seeds (no./%)	118/39.3	125/41.7^a^	243/40.5	234/78.0^d^
Dead & diseased seedlings (no./% of germinated)	84/71.2	86/68.8^a^	170/70.0	0/0^d^
Seedling length (mm, mean ± SD)				
– stem	24.4 ± 2.0	28.1 ± 2.2^b^	26.3 ± 1.4	41.0 ± 2.4^d^
– main root	4.7 ± 0.4	6.7 ± 0.5^b^	5.7 ± 0.4	18.9 ± 1.4^d^
whole	29.1 ± 4.6	34.8 ± 3.0^b^	32.0 ± 2.6	59.9 ± 8.6^d^
Seedling dry weight (mg, mean ± SD)	0.60 ± 0.1	0.65 ± 0.03^a^	0.61 ± 0.03	1.9 ± 0.1^d^
Re-isolation of the fungus (no./%)	80/95.2	79/91.9	159/93.6	—

^a^Difference between the tested isolates (N1 *vs*. N2) non-significant.

^b^Difference between the tested isolates (N1 *vs*. N2) significant at *p* < 0.05.

^c^Difference between both tested isolates and control (pooled N1 + N2 *vs*. control) non-significant.

^d^Difference between both tested isolates and control (pooled N1 + N2 *vs*. control) significant at *p* < 0.0001.

^e^Evaluated visually, scoring system: score 4: mycelium covers surface of an inoculated chip by >75%; score 3: 50–75%; score 2: 25–50%; score 1: <25%; score 0: mycelium absent.

#### Saplings of *Abies sibirica*

**Figure 5:**
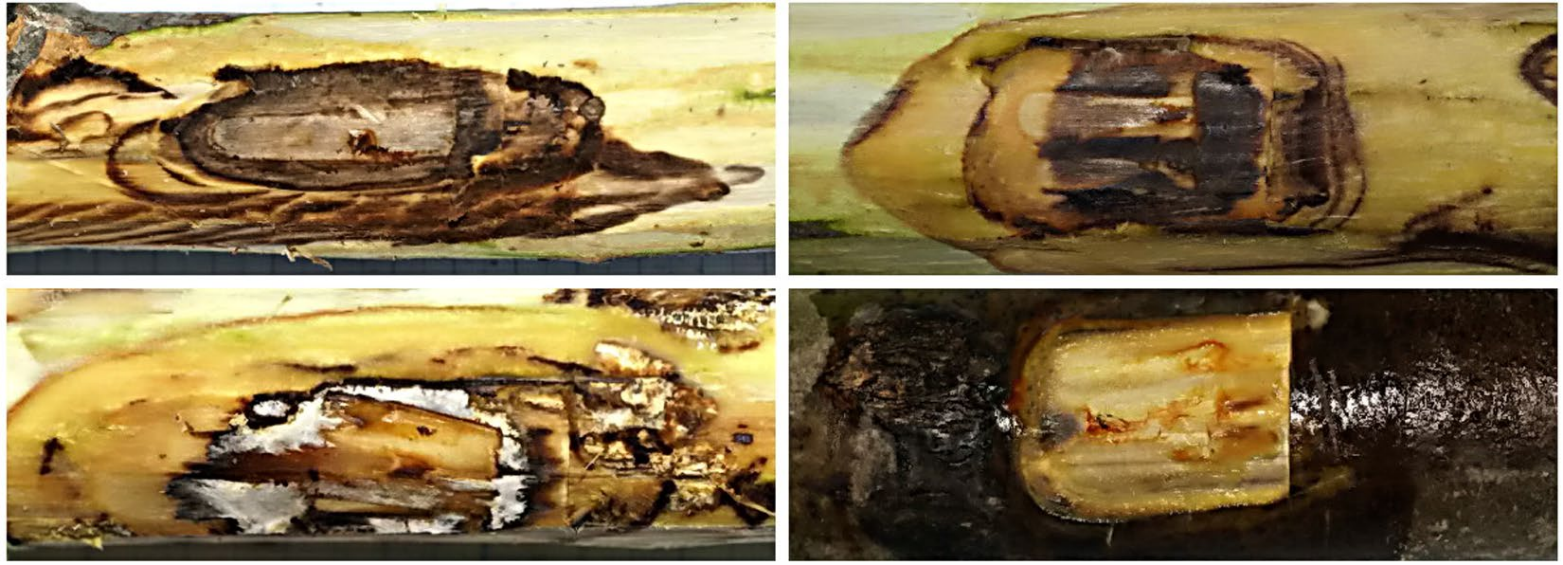
Extent of necrosis in cambial zone beyond inoculation wounds after the removal of an inoculation chip. Right bottom picture shows control. Diameter at wounding/inoculation point approx. 10 mm.

When pooled, the proportion of diseased/dead saplings inoculated with the isolates of the anticipated pathogen (*Corinectria* sp. X, strains N1 and N2) was significantly higher than that observed among saplings in the control set (53 out of 60 *vs*. 2 out of 30; *p* < 0.0001). The same results of the similar significance was obtained when the proportions of diseased/dead in respective set of each isolate were compared with the control in separate tests (for it was N1, 27 out of 30, and for N2, 26 out of 30 *vs*. control). However, the proportion of dead saplings observed in inoculations and the control was non-significant (12 out of 60 *vs*. 2 out of 30). Respective comparisons between saplings inoculated with N1 and N2 in each case were non-significant. Phloem necrosis in control saplings has not been observed, while in the inoculated ones it comprised on average 44.4 mm in longitudinal direction, and 17.1 mm in lateral cross-section (in summary, outside inoculation boundaries) (Fig. 5). Yet, in this respect the differences in extent of caused necrosis between the tested isolates (N1 *vs*. N2) were significant at *p* < 0.05. Saplings, girdled by the necrosis were not observed. Re-isolation of inoculated strains from the saplings was in most cases (76.7%) successful.

#### Seeds and seedlings of *Abies sibirica*

There were no statistically significant differences in germination incidence between seeds sawn into sand containing chips pre-inoculated with mycelia of anticipated pathogens and the controls (respectively, 402 out of 600, or 67%, *vs*. 210 out of 300, or 70%). Yet, following the germination, the proportion of furthermore observed dead/diseased seedlings among those subjected to pre-inoculated chips *vs*. controls, was markedly significant (respectively, 334 out of 402, or 83.1% *vs*. 18 out of 210, or 8.6%; *p* < 0001). In those respective comparisons between strains N1 and N2, the differences were non-significant. Visually assessed chip coverage by inoculated mycelium at the end of the experiment in most cases constituted 25–50%. Re-isolations of the inoculated strains from both the chips and seedlings were in most cases successful, i.e. 87.5% and 90.7%, respectively.

#### Seeds and seedlings of *Picea abies*

**Figure 6:**
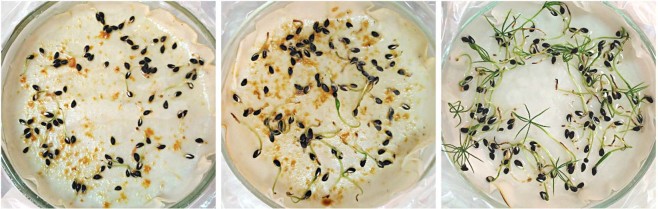
Seeds of *Picea abies* after 14 days, a-priori soaked in cultural filtrate, and placed to germinate in sterile 9 cm Petri plates with moist filter paper. A picture on the right shows control.

The difference in germination incidence between seeds subjected to conidial suspension/metabolites and controls was statistically significant at *p* < 0.0001 (respectively, 243 out of 600, or 40.5%, *vs*. 234 out of 300, or 78%). Following the germination, the proportion of furthermore observed dead/diseased seedlings among those subjected to metabolites *vs*. controls, was also markedly significant (respectively, 170 out of 243, or 70% *vs*. 0 out of 234; *p* < 0001) (Fig. 6). In those respective comparisons between strains N1 and N2, the differences were non-significant. The differences in length of the germinated seedling (stem, main root, and as a whole) were in each comparison of inoculation *vs*. control highly significant (e.g. in whole length, 32.0 *vs*. 59.9 mm; *p* < 0.0001), but were also significant between the two inoculation sets (N1 *vs*. N2, 29.1 *vs*. 34.8 mm; *p* < 0.05). Similarly, also the differences in seedling dry weight between inoculations and controls were highly significant (respectively 0.6 *vs*. 1.9 mg; *p* < 0.0001). Re-isolation of the fungus from diseased seedlings was successful in 159 cases of 170 attempts (93.6%).

## Discussion

Despite the complexity of the present study, it produced rather simple results leading to several straightforward conclusions, and certain guidelines for the future research. It demonstrated that, (i) from canker wounds on branches and stems of re-growth *Abies sibirica* pure cultures of *Acremonium*-like anamorph were regularly isolated; (ii) those isolates belong to ascomycetous fungi from the genus *Corinectria*, and so far in the present study two distinct species have been detected, neither of which is known to science, and currently remain unidentified; (iii) at least one species of those (represented by isolates N1 & N2), on its own has the capacity to kill *A. sibirica* saplings and seedlings, but also demonstrates certain capacity to be pathogenic to *Picea abies* e.g. by reducing growth and killing its seedlings. It provided the following implications.

### The identity

The species must be described. In this respect, more fungal material (e.g. isolates and sporocarps) needs to be collected and provided scientific names, as e.g. this was done by Gonzalez and Chaverri^2^ for a number of so far known *Corinectria* spp. from global collections. In support, the molecular identification and phylogenetic analysis provided the evidence that the isolated fungi represent new species within the genus *Corinectria* due to distinct placement and well supported separation from the other closely related fungal species (Fig. 4).

### The origin

The question must be answered: are those native, until recent cryptic *Corinectria* species of this geographic area, pathogenic activity and spread of which have been triggered by the climate change and/or associated activities of other pathological factors known in the area, as e.g. root rot caused by *Armillaria borealis* Marxm. & Korhonen, and *Heterobasidion parviporum* Niemelä & Korhonen^4^, and following attacks by *Polygraphus proximus* Blandford^5^; or, are those invasive fungi, with yet unknown origin and modes of invasion, e.g. spread by plant material trade, or spread by airborne spores, insect-vectored?

### The risk

Generally, *Corinectria* (prev. *Neonectria*) spp. are well known to be canker pathogens of a wide spectrum of conifer species from a broad geographic range. For example, *Corinectria*/*Neonectria* spp. (in reports before 2017 identified as *Nectria*/*Neonectria fuckeliana*, but currently, in light of results of subsequent molecular studies, those citations should be referred to as *N. fuckeliana sensu lato*) have been frequently reported as a cause of canker disease (and in some cases on nursery stock) of *Picea* spp., *Abies* spp., *Larix* spp. and *Pinus* spp. in Europe^6–9^, North America^10–12^, New Zealand^3,13,14^, and Chile^15,16^.

Therefore the following question must be answered: to which extent Siberian *Corinectria* pose a risk to health of other conifer species? In order to do this, inoculation tests with saplings of possibly many conifers (incl. exotic species, as many of those are grown, e.g. in urban environments) have to be accomplished. In particular, this is of crucial importance for Europe, as here geographic range of *A. sibirica* and *P. abies* to a large extent overlap (Fig. 1), and there extensive trade of internal movement of wood, esp. within Russia. Consequently, this part of the work should be done in close collaboration with the colleagues in Russia. In particular, in this respect one must keep in mind an example of rather recent (around year 2000) invasion to Europe of the Emerald Ash Borer (*Agrillus plannipennis* Fairmare), which presumably has been imported with *Fraxinus* wood from Siberia to Moscow, and subsequently spread in all directions on a broad geographic scale, devastating ash populations in cities and forest land, and currently is approaching eastern EU borders^17^. The lessons from the past must be learned.

## Materials and methods

### Field sampling

Investigated site was located in Central Siberia, about 15 km west of Krasnoyarsk (N56°01′41.46″; E92°34′39.30″). Samples were collected from five *A. sibirica* trees exhibiting typical symptoms of the disease. The trees were approximately 30–50 years-old, 6–13 m high, and 8 –14 cm in dbh. The sampling targeted parts of branches and stems representing discrete cankers (Fig. 2A–C). Symptomatic parts of the branches were cut off using a handsaw. Cut branch sections were approx. 4 cm long and 2 cm in diameter. Samples from stems were taken directly as pieces of diseased bark and wood using a chainsaw and an axe. Approximate size of stem samples taken in the field was 10 × 5 × 3 cm. Each sample was individually placed into a plastic bag and transported to the laboratory. A total of 64 samples was collected from three arbitrary categorized symptomatic canker types (Table 1).

### Isolation of mycelia and morphological identification

In the laboratory, phellem was removed from the surfaces of cankers, and 20 × 5 mm size slivers of phloem/xylem were aseptically cut off across the canker-symptomatic areas, and sampled using ethanol + flame sterilized scalpel and forceps, taking 5–7 slivers per canker. Numbers of sampled cankers from each symptomatic category are shown in the Table 1. The slivers were surface sterilized in 70% ethanol, followed by rinsing in sterile water, all for 30 seconds per step, placed on 2% malt extract agar (MEA) and incubated at 20 °C in the dark. Mycelia that emerged from the samples were cut off from uniform morphologically discrete hyphal colonies and subcultured onto fresh MEA.

Morphological observations of the colonies in culture were carried out on isolates grown on potato-dextrose agar (PDA) (Fig. 2D), carrot agar (CA) (Fig. 2E), MEA (Fig. 2F), carnation-leaf agar (CLA), and slow nutrient agar (SNA) for 4 wk. at 20 °C with alternating 12 h/12 h mixture of cool-white fluorescent + UV-light/darkness. The colony growth rate was measured on 9 cm Petri dishes containing PDA, 5 mm deep, inoculated with a 10 mm diameter mycelial plug after 14 days. Microscopic observations of the isolates were made using an Olympus CX41 microscope (Olympus Co., Tokyo, Japan) and a scanning electron microscope Hitachi SU3500 (Hitachi, Tokyo, Japan). Fourteen pure cultures showed morphological characters typical for *Acremonium-*like (*Corinectria*) anamorphs (Table 1; Fig. 2D–F; Fig. 3)^2,3^, and four of those were included into further molecular analyses.

### Molecular identification and phylogenetic analyses

DNA isolation from the four Siberian cultures was done using the CTAB method^18^. Briefly, fungal mycelium of each strain was collected from Petri dishes into individual 1.5 ml centrifugation tubes and together with glass beads homogenized by vortexing. To each tube, 1 ml CTAB buffer (3% cetyltrimetylammoniumbromide, 2 mM ethylenediamine tetraacetic acid, 150 mM Tris–HCl, 2.6 M NaCl, pH 8) was added, and the samples were incubated at 65 °C for 1 h. After centrifugation, the supernatant was sequentially extracted with an equal volume of chloroform, centrifuged for 7 min at 13,000 rpm, precipitated with 1.5 volumes of 2-propanol, washed with 70% ethanol, dried and redissolved in 50 μl ddwater^18^. Concentration of DNA in individual samples was determined using a Nanodrop spectrophotometer (Thermo Fisher Scientific, Waltham, MA, USA).

The fragments amplified were α-actin (ACT), β-tubulin (BTUB), the complete internal transcribed spacer of rDNA (ITS) and translation elongation factor 1α (TEF1). Primers and protocols for amplification were as in González and Chaverri^2^. The corresponding reference sequences for *Corinectria* species, *Neonectria faginata*, *N. neomacrospora*, and *Thelonectria discophora* were downloaded from the GenBank and are listed in Table 2. The sequences of each genetic marker were aligned using ClustalW and manually adjusted in BioEdit^19^. A partition homogeneity test in PAUP* 4.0a166^20^ has been run to check if there were no conflicts among different loci. As no significant conflict was detected (*p* > 0.45), DNA sequences were concatenated into a single supermatrix. The JModeltest^21^ was used to determine the nucleotide substitution model for the combined dataset, which was GTR + G. The Bayesian Inference (BI)-based phylogenetic approach was performed in MrBayes v3.2.7^22^. Support for nodes was estimated using 1,000 fast bootstrap (BS) pseudo-replicates in PAUP Maximum likelihood (ML) analyses and by the posterior probabilities of nodes calculated after convergence of two independent MrBayes runs each of two chains run to 2 × 10^6^ generations, sampling every 1,000 generations with a consensus tree computed after a final burnin of 20% of the trees. Bayesian posterior probabilities ≥0.95^23^, and ML bootstrap support ≥70% were considered to be significant. Sequences of Siberian isolates generated were deposited in GenBank (Table 2).

### Pathogenicity tests

Two isolates of *Acremonium-*like (*Corinectria*) anamorph N1 (NfP5.7) and N2 (NfSp3.4) were tested for pathogenicity. Prior to the experiment, the isolates were stored in the lab on PDA for one year under 4-6 °С. Pathogenicity tests involved a total of 90 saplings (30 and 30 for each respective isolate, and 30 for control) and 900 seeds/subsequently seedlings of *A. sibirica*, and 900 seeds of *P. abies* (here, in a similar setting for both tree species, 300 + 300 for each isolate, and 300 for control) (Table 3). Control plants/seeds were subjected to similar test procedures, but in the absence of the anticipated *Acremonium-*like *(Corinectria*) pathogen. Seed material was collected from randomly selected trees of the respective species. Comparisons of recorded data between inoculated/control sets were made using chi-squared tests (for proportions) and *t*-tests (for means).

#### Saplings of *Abies sibirica*

In this part of the work, methodological approach followed studies by Bakys *et al*.^24,25^, where artificial inoculations were used to test pathogenicity of fungal isolates to saplings of *Fraxinus excelsior* L. Saplings of *A. sibirica* (age 7–10 years, height 40–45 cm, diameter at root collar 10–12 mm) were inoculated with wood chips, size 20 × 5 × 3 mm (length × width × thickness) pre-colonized by mycelium of a tested isolate. For this, sterilised chips were placed on a pure culture for approx. 4 weeks. Bark wounds were cut on stems approx. 50 mm high from the soil, the inoculated chip placed on a wound and wrapped with Parafilm (Bemis Company Inc, Neenah, WI, USA). Control saplings were of the same characteristics and were treated in a similar manner, but using sterile chips. Each sapling was planted in a container of 20 litres sterilised sand, placed in a climatic chamber at 25 °C and subjected to periodic humidification under natural light for three months (March, April, May). The following data were recorded.

1. Incidence of dead and diseased saplings per isolate-inoculation set, and control set.
2. The viability of bark and phloem, scored as either visually healthy (bark tightly attached, phloem colour light-green), or diseased/necrotic (bark detached, phloem colour brown to black).
3. Extent of necrosis in cambial zone beyond inoculation wound (Fig. 5). It was measured with a flexible ruler: longitudinal extension from the inoculation boundary both upwards and downwards, and lateral at the cross-section to both sides, left and right, making a total of four measurements per inoculation wound.
4. Re-isolation frequency of inoculated fungus from dead and diseased saplings. At first, presence/absence of the mycelium was visually evaluated at 5-10 mm distance above from the inoculation point by removing fragment of bark with a sterile scalpel and placing it into a “wet camera” (Petri dish containing double layer of sterile filter paper soaked in distilled water), and subsequently checking for fungal growth with the use of a microscope. When present, a fragment of mycelium was removed using sterilised needle and placed on CA and MEA to record an appearance/morphology of mycelial growth characteristic for inoculated *Acremonium-*like (*Corinectria*) isolate (Fig. 2E, F; Fig. 3). All inoculated saplings (30 + 30) were subjected to re-isolations (Table 3).

#### Seeds and seedlings of *Abies sibirica*

This part of the work followed modified methodological approach used in studies by Lazreg *et al*.^26^, Alghuthaymi *et al*.^27^, and Güney & Güldür^28^. Seeds of *A. sibirica* were sterilised in 0.5% dilution of KMnO_4_ for 120 min, washed with sterile water, dried in-between layers of sterile filter paper and sawn into plastic 350 ml volume containers, size 107 × 82 × 70 mm (length × breadth × height), filled with heat-sterilised sand intermixed with wood chips of size 20 × 10 × 3 mm. The chips were sterilised and pre-colonised by the mycelium by placing them on a pure culture of the inoculum fungus for approx. 4 weeks. At first, a bottom of each container was covered by a 5 mm thick layer of wet sterile sand, on the surface of which were placed pre-colonised chips, fifteen in each container. Placement scheme was in a straight-angled rows 5 × 3: five chips in three rows; distance between chips in a row, approx. 1.5 mm, distance between rows approx. 12 mm. They were covered by 2 cm thick layer of wet heat-sterilised sand. Seeds were sown on sand surface and afterwards thinly shed by sand, 100 seeds per container, making in total three containers per each of the two tested strains of the fungus (300 seeds/strain, in total 600). Three control containers were processed in a similar manner, but included sterilised chips (Table 3). All containers were covered by caps (for approx. 25 days, after which regular germination has appeared and caps were removed), placed in a climatic chamber at 25 °C and subjected to periodic humidification under natural light for three months (under similar regime as for saplings, see above). The following data were recorded.

1. Frequency of seed germination.
2. Incidence of dead and diseased seedlings.
3. Extent of chip surface coverage by inoculated mycelium (exhibiting typical yellow – orange color) by the end of the experiment. It was evaluated visually, according to the following scoring system. Score 4: mycelium covers the surface of an inoculated chip by >75%; Score 3: 50–75%; Score 2: 25–50%; Score 1: <25%; Score 0: mycelium absent (as, e.g., in controls that also have been checked). Each inoculated chip has been subjected to such evaluation, meaning 15 from each container, thus 45 for each isolate (Table 3).
4. Re-isolation frequency from inoculated chips. This was done by cutting off (sterilised scalpel) approx. 5 × 5 mm size fragment of a chip, and then transferring (sterilised forceps) cut wood sample to a “wet camera”. After 3–6 days, emerging mycelia from those were individually transferred (sterilised needle) to Petri dishes containing MEA. From each inoculated container, eight chips were subjected for re-isolation, making 24 for each isolate, a total of 48 (Table 3).
5. Re-isolation of the pathogen from dead and diseased seedlings. The seedlings were extracted from the container soil with roots, individually washed under the running water, then submerged for 1 min in 5% dilution of Ca-hypochlorite, washed in sterile water, dried with sterilised filter paper, cut (using sterilised scalpel) into 15–20 mm fragments, which were placed into Petri dishes onto MEA and CA. All dead and diseased seedlings from each container were subjected to re-isolation, 169 for the isolate N1 and 165 for N2, making a total of 334 (Table 3).

#### Seeds and seedlings of *Picea abies*

This part of the work followed modified methodological approach used in the study by Alghuthaymi *et al*.^27^. Each of the same tested two *Acremonium-*like isolates were grown separately in two separate sets in liquid Norkrans media during 14 days at 25 °С. In order to test for germination rates and subsequent condition of eventually germinating seeds, 300 + 300 seeds of *P. abies* were soaked in the cultural filtrate of each respective strain for 24 hours, and placed to germinate in sterile Petri plates with double layer of moist filter paper (“wet cameras”) for 21 days at a temperature of 25 °C. For control, 300 seeds were soaked in sterile water and subjected to the germination test in a similar way (Fig. 6). The following parameters were recorded.

1. Frequency of seed germination.
2. Incidence of dead and diseased seedlings.
3. Length of a seedling stem and main root.
4. Seedling weight. This was done by individually removing of survived seedlings from”wet camera”, dried till air-dry permanent weight, and then weighted.
5. Re-isolation of inoculated fungi from seedlings was performed by individually washing those under the running water, then submerging for 1 min in 5% dilution of Ca-hypochlorite, washed in sterile water, dried with sterilised filter paper, and placing those into Petri dishes onto MEA and CA.

## Data Availability

The datasets generated and/or analyzed during the current study are available from the corresponding author on request.
